# The impact of behavioral and environmental factors on cancer mortality in G7 countries: a 20-year ecologic study

**DOI:** 10.3389/fpubh.2025.1601796

**Published:** 2025-10-07

**Authors:** Yezdan Caglar, Macide Artac Ozdal

**Affiliations:** Department of Health Management, Faculty of Health Sciences, European University of Lefke, Northern Cyprus, Türkiye

**Keywords:** cancer mortality, G7 countries, modifiable risk factors, public health policy, economic, analysis, lifestyle factors

## Abstract

**Background:**

The Cancer death rates prevails as a critical challenge on health systems globally. Whilst various factors such as economics and lifestyle factors are predicted to be influential, it is important to explain these relationship scientifically to develop targeted public health interventions.

**Objectives:**

The investigation within this study concerns the interconnections between the rates of death arising from Cancer and pivotal lifestyle and economic factors of the populations within the economically advanced countries of the G7 (Germany, the United Kingdom (UK), The United States of America (USA), Canada, France, Italy, and Japan) over 20 years, from the year 2000 until 2020.

**Methods:**

The data used in this study, including GDP (in United States Dollars USD - $) influence, obesity rate, pollution levels, population size, the prevalence of smoking, and the cancer death rates were collected from World Bank and Organization for Economic Co-operation and Development (OECD). Descriptive statistics, multiple linear regression, ANOVA, the case Processing Summary, and Principal Component Analysis were carried out to determine the distribution of data and interrelationships between independent and dependent variables.

**Results:**

The findings of the study disclosed that the variables explained 58% of the variance in cancer mortality (*R*^2^ = 0.580). Definite connections between smoking prevalence, years of life lost, levels of obesity, the level of pollution, and the cancer death rate were established.

**Conclusion:**

The study emphasizes the importance of targeting modifiable risk factors such as smoking, pollution, and obesity in cancer prevention and management. It highlights the importance of public health strategies focused on reducing these risk factors through targeted interventions. Additionally, equitable healthcare distribution must be considered in shaping effective policies to reduce cancer mortality.

## Introduction

The death rate deriving from Cancer is a concern of great importance to public health as various environmental management factors are seen to exert influence on levels of occurrence (1). Ecological management focuses on sustainability by addressing environmental issues through strategic decision-making and actionable policies. It involves mitigating risks, allocating funds for ongoing improvements, and addressing past ecological damage (2).

This study categorizes environmental factors influencing public health into three main sub-factors. The first is attributed to national public authorities and the lack of strength of their investment strategies to have a relevant influence on GDP in United States Dollars (USD - $), sequentially exposing citizens to a negative consequence. The second sub-factor is the regional environmental occurrences, such as the pollution of the air and soil contamination from chemicals, in addition to the availability of adequate clean water. The final sub-factor is regarding the chosen behavior of individual citizens, namely the use of tobacco products and obesity levels. Furthermore, alternative forced determinants such as unstable physical and psychosocial impositions with origins in both personal and work-life tensions (3).

Advances over time have enhanced understanding of how environmental factors influence the risk of cancer, thus underscoring their global domination on Cancer prevalence (4). Public health continues to endure the challenges presented by Cancer, which exists as an initiator of premature death worldwide, exacerbated even more by the pattern of increasingly unhealthy lifestyle habits. Studies suggest that 40% of cancer cases in affluent countries are preventable through lifestyle changes, including increased physical activity, healthier diets, and reduced smoking and alcohol use (5). Modern advancement in the ability to enhance the speed of detection and thereafter rapid clinical treatments with supportive care has led to reducing the mortality rate of Cancer patients. Since 2019, it has been noted that the relative 5-year survival rate for a combination of Cancers has reached a figure of around 68%, prompting the rise of survival to more than 16.9 million Cancer sufferers in the United States (USA). Regrettably, many of these survivors are unable to return to normal life, often enduring long-term health problems along with a lower quality of life than previously experienced. Cost-effective interventions are key to confronting the ongoing burden of Cancer as well as battling against the rate of mortality caused by the illness. The crucial risk factors of Cancer, which are modifiable, enabling higher survival prospects, include the absence of a healthy diet, a higher than advisable body mass, inadequate physical exercise, and use of tobacco products. Past epidemiological studies have typically presumed that lifestyle behavior at one point in adulthood is a lifetime habitual behavior, accepting lifestyles are constant. Prevention strategies to fight Cancer aim to encourage behaviors that are sustainable and flexible enough to withstand behavior variability throughout life. The total significance of life changes during adulthood has still not been fully understood (6). The largest impact on public health is a habitual unhealthy lifestyle, which includes excess eating and drinking of alcohol, lack of exercise, poor quality diet, and smoking. Over 50% of the total diagnosed Cancer cases and over 40% of cardiovascular illnesses, including heart disease, are considered to be in some form associated with factors relating to detrimental lifestyles (7).

Between the years 2005 and 2015, cancer occurrence has significantly risen by 33%. This has been mainly attributed to the growth and aging of populations. Despite this rise, it is estimated that modification of lifestyles could save more than a third of the lives lost due to Cancer. A key feature of global Cancer control is primary prevention, adjustment of certain factors could change outcomes, including smoking (21%), lack of physical exercise (2%), the use of alcohol (5%), obesity (2%) and the consumption of fresh fruit and vegetables (5%) (8).

The purpose of this research is to evaluate the association between Cancer death rates and various environmental and lifestyle factors, including GDP in USD, pollution, population size, smoking and obesity in the countries of the G7, namely Germany, the United Kingdom (UK), the USA, Canada, France, Italy and Japan. The study will explore how the Cancer death rates are affected by the increasing or decreasing trends in GDP in USD, obesity rates, the effects of pollution, population size, smoking rates, and life lost in the particular countries.

## Materials and methods

This study used an ecological study design to assess selected environmental and lifestyle factors on the Cancer mortality rates. Lifestyle habits, such as obesity rates and tobacco use and environmental factors, including pollution, population size, and life expectancy were studied in relation to Cancer mortality rates in G7 countries, including Germany, the UK, the USA, Canada, France, Italy and Japan. The definitions of the data used are provided in the Table 1. The data for the study were collected from the website of Organization for Economic Co-operation and Development (OECD) and World Bank. The study focused on G7 countries, since they are the countries that have more consistent advancement in the world and long-term data for the period of 2000–2020 were well-established due to their comprehensive health data systems. Nations not included in the G7 group were excluded from the analysis, because of inconsistency in their levels of social and economic evolution, reflecting to the poor quality of the data available for them.

**Table 1:** The definitions and explanations of the dependent and independent variables included in the study.

Variables	Definitions
Dependent variable	
Cancer deaths	Deaths from cancer are the mortality rate resulting from all types of malignant neoplasms. Mortality rates are based on the number of deaths registered in a country in a year divided by the size of the corresponding population. The rates have been age-standardised using the direct method of standardisation to the OECD population to remove variations arising from differences in age structures across countries and over time.
Independent variable	
Smoking	Smokers are the population aged 15 years and over who report that they smoke tobacco every day. International comparability is limited due to the lack of standardisation in the measurement of smoking habits in health interview surveys across OECD countries. There is variation in the wording of the question, the response categories, and the related administrative methods.
Population size	The population is the number of people who live in a country. It counts the resident population, defined as all nationals present or temporarily absent from the country, and aliens permanently settled in the country. The population includes the categories: national armed forces stationed abroad; merchant seamen at sea; diplomatic personnel located abroad; civilian aliens resident in the country; and displaced persons resident in the country. Populations excluded are namely foreign armed forces stationed in the country; foreign diplomatic personnel; and civilian aliens temporarily resident in the country. For countries with overseas colonies, protectorates, or other territorial possessions are generally excluded.
Pollution	Estimated annual age-sex specific disability adjusted life years (DALY) in millions attributable to environmental factors, including Air Pollution, Water Pollution, Greenhouse Gas Emissions, Land Pollution and Solid Wastes that are caused by vehicle exhaust, factories, dust, pollen and natural processes, such as volcanoes and wildfires.
Potential years of life lost	Potential years of life lost is a summary measure of premature mortality, providing an explicit way of weighting deaths occurring at younger ages, which may be preventable. The calculation involves adding deaths occurring at each age and multiplying this by the number of remaining years to live up to a selected age limit (75 years old is used in OECD Health Statistics). To assure cross-country and trend comparison, the potential years of life lost are standardised for each country and each year.
Gross Domestic Product (GDP in USD)	Gross Domestic Product (GDP) is determined according to the expenditure approach. In the expenditure approach, the main components of GDP are including final consumption expenditure of households and non-profit institutions serving households (NPISH), plus final consumption expenditure of General Government, plus gross fixed capital formation (or investment), and plus net trade (exports minus imports).
Obesity	Overweight or obese population is the share of the population aged 15 years and older with excessive weight presenting health risks because of the high proportion of body fat. Based on the World Health Organization (WHO) classification, adults with a body mass index (BMI- weight/height^2^) from 25 to 30 are defined as overweight, and those with a BMI of 30 or over as obese. Data is recorded both for “self-reported” data (estimates of height and weight from population-based health interview surveys) and “measured” data (precise estimates of height and weight from health examinations).

### Data analysis

In this study, data were analyzed using the statistics tools, namely SPSS Version 22 and R Version 4.4.2. The data was first analyzed using descriptive statistics, analyzing the characteristics of the data. The associations between the dependent and independent variables were then analyzed using Correlation analysis, Multiple Linear Regression analysis ANOVA, the case Processing Summary, and Principal Component Analysis.

### Descriptive statistics

The data set was summarized by descriptive statistics, which offered insight into principal measures such as the mean, standard deviation, and distribution of variables, including levels of obesity, the amount of pollution, the size of the population, the prevalence of smoking, and the expected life length. The data were tested for normality using descriptive statistics. Since skewness and kurtosis values were within the acceptable ranges (−2 and +2 for skewness and −1 and +1 for kurtosis values), all the variables were considered as normally distributed (9). This course of action produced a comprehensive overview of the data set, which identified central tendencies and variations in cancer mortality rates.

### Correlation analysis and multi-collinearity

Correlation analysis was used to identify relationships between cancer mortality and the independent variables. Correlation measures the degree of relationship between two variables. It ranges from −1 to 1, where 1 indicates a perfect positive correlation and −1 indicates a perfect negative correlation. Correlation is beneficial for exploring the relationship between two variables and identifying potential predictors (10). Variance Inflation Factors (VIF) were calculated to assess multi-collinearity. Multi-collinearity is the occurrence of high correlations between two or more independent variables in a multiple regression model. This method is used to determine how effectively each independent variable can be used to predict the dependent variable in a statistical model. Multi-collinearity can lead to misleading results, with wider confidence intervals that produce less reliable probabilities for the effect of the independent variables in a model (11). Principal Component Analysis (PCA) was used to address multi-collinearity and reduce dimensionality, retaining variables that significantly influenced cancer mortality. As a result of the PCA analysis, the components obtained were used to produce a biplot graphic that allowed the presentation of the proximity of the variables to each other.

### Regression analysis

Both multiple linear regression and Poisson regression models were used to explore relationships between cancer mortality and independent variables. Regression analysis is an analysis method used to measure the relationship between two or more quantitative variables. Since more than one variables were assessed in this study, multivariate regression analysis was employed (12). Poisson Regression Analysis is a statistical method used to estimate the number of times a specific event will occur within a period for independent variables (13). The Poisson model was chosen for its suitability in analyzing rate data, providing robust insights into the effects of smoking prevalence, pollution levels, and life expectancy.

### ANOVA and PCA

A One-Way ANOVA tested for statistically significant differences among groups, revealing non-uniform impacts of variables like smoking, pollution, and obesity across G7 countries. One-way ANOVA analysis is a tool used to test whether there is a statistically significant difference between the means of independent groups (14). PCA further simplified the dataset, highlighting smoking, pollution, and life expectancy as the most influential factors in cancer mortality.

The combination of advanced statistical techniques, sensitivity analyses, and temporal considerations ensures a robust and comprehensive methodology to appreciate the factors dominating the cancer death rate throughout the G7 countries.

Confirmation of the statistical significance of the model (*p* < 0.05) emerged from the ANOVA test, accentuating that characteristics of lifestyle and environmental factors are not consistent influences on the death rate of cancer sufferers throughout the countries of the G7. The variance between the means of individual groups was significantly great to indicate that at the minimum, there is one determinant (the obesity level, pollution effect, the GDP, population size, or smoking generality) that notably exerts influence on the rate of cancer mortality in comparison to the remaining factors.

## Results

Significant findings relating to the dominance of the components attributed to lifestyle and environmental factors along with their significance on cancer patient mortality rates within the G7 countries over 20 years (2000 to 2020) were produced from the data analysis of this study.

147 total data points are presented in this study without any data absence. Consequently, the data absence denotes the nonexistence of a response for observation purposes. However, confusion with a zero value should be avoided. Confirmation of the completion of data concerning death deriving from cancer, the GDP in USD, the effects of pollution, the level of tobacco product use, and the loss of life from the statistical test analysis. Consequently, data relating to the research displayed a non-existence of missing values in the results obtained.

The descriptive statistics produced an evaluation of the crucial set of values involved in the study. Within the countries of the G7 during the period of research, the mean rate of cancer-caused deaths was in the region of 224.915, with a standard deviation of 21.7615 (Table 2). The means of death caused by Obesity levels and the effects of pollution saw specifically elevated means, which demonstrated the extensiveness experienced in the prosperous countries. Exhibiting a marked variance in the sizes of a population, the prevalence of using tobacco products, in addition to the conjecture of length of life, illustrated the demographic diversity of the G7 countries. (Note: as the correlation of death caused by cancer was negligible, GDP in USD was not used.)

**Table 2:** Descriptive statistics of dependent and independent variables, namely cancer death rates, pollution effect, smoking, population size, GDP in USD and obesity in G7 countries between the years of 2000 and 2020.

Variables	Mean	Std. Deviation	*N*
Death cancer	224.915	21.7615	147
Obesity	53.836	13.3388	147
Pollution effect	294.456	127.3719	147
Population	105.422	87.3436	147
Smokers	20.625	5.0104	147
Life lost	4684.673	1077.7289	147

In Table 3, the *R*-squared and Durbin-Watson statistics are important. The *R*-squared is 0.580, meaning that the research model’s independent variables explain 58% of the variance in the dependent variable. The remaining 42% is due to other factors. The Durbin-Watson statistic is a test for autocorrelation in the residuals of a regression model. The DW statistic ranges from zero to four, with a value of 2.0 indicating no autocorrelation. Values below 2.0 indicate positive autocorrelation. The statistical results show positive autocorrelation.

**Table 3:** Poisson regression model associating cancer death rates with dependent and independent variables, namely cancer death rates, pollution effect, smoking, population size, GDP in USD and obesity in G7 countries between the years of 2000 and 2020.

Model	*R*	*R* square	Adjusted *R* square	Standard error of the estimate	Durbin-Watson
1*	0.762	0.58	0.565	14.3519	0.327

Model was constructed by including cancer death as dependent variable and smoking status, obesity, population size, pollution effect and GDP in USD as independent variables (predictors).

In this instance analysis of factors deriving from the environment and a dependent variable was performed using Multiple linear regression, it can examine the linear relationship betwixt a minimum of two independent variables (15). In a similar context, the linear relationship between the dependent variable (the cancer-causing death rate) and several independent variables was examined (GDP in USD, the existing level of obesity, effects originating from pollution, the population size, and the prevalence of smokers). Data analysis, estimation, and understanding of causal relationships were the purposes of this method, to determine the influences of independent variables on the dependent variable. The determination of the effect of variables such as smoking tobacco products and obesity on the cancer mortality rate is established by multiple linear regression. Table 4, namely the coefficient table, exhibits the influence of the independent variables on the dependent variable, cancer death. The unstandardized coefficients which are as follows:


$$\begin{array}{c} \text{Cancer Death}=115.528+(\;\text{obesity}\times 1.103)\\ +(\mathrm{pol}\_\text{effect}\times 0.010)+(\mathrm{GDP}\times -4.10\mathrm{9E}-6)\\ +(\text{population}\times -0.069)+(\text{smokers}\times -2.834). \end{array}$$

**Table 4:** Linear relationship between dependent and independent variables in G7 Countries. (2000–2020).

Model	Unstandardized coefficient			Collinearity statistics	
	B (Significance)	Std. Error	*T*	Tolerance	VIF
Constant	115.528 (*)	9.848			
Obesity	1.103 (*)	0.103	0.676	0.752	1.33
Pollution effect	0.010 (*)	0.011	0.058	0.747	1.339
GDP USD	−4.11E-06 (*)	0.000	−0.219	0.7	1.429
Population size	−0.069 (*)	0.016	−0.277	0.737	1.357
Smokers	2.834 (*)	0.334	0.653	0.503	1.988

^*^p-value<0.001.

How each independent variable affects the rate of death from cancer is illustrated in this equation. For example, a decrease in the death of cancer by 0.206 units is seen when an increase in the obesity variable occurs, whilst a rise in the effects of pollution denotes an increase in death deriving from cancer by 0.048 units. Since the measurement of multicollinearity in a regression analysis is key for model fit, the Variance Inflation Factor (VIF) value is of crucial importance. The Multi-collinearity occurs upon an intercorrelation of two or more independent variables found in a multiple regression model, considering the possibility of negative impact of this intercorrelation on the regression results. An indication that multi-collinearity exists is generally exhibited by either a VIF above 4 or a tolerance below 0.25; accordingly, additional research is necessitated. Hence, a similar situation for the independent variables was not detected.

**Figure 1:**
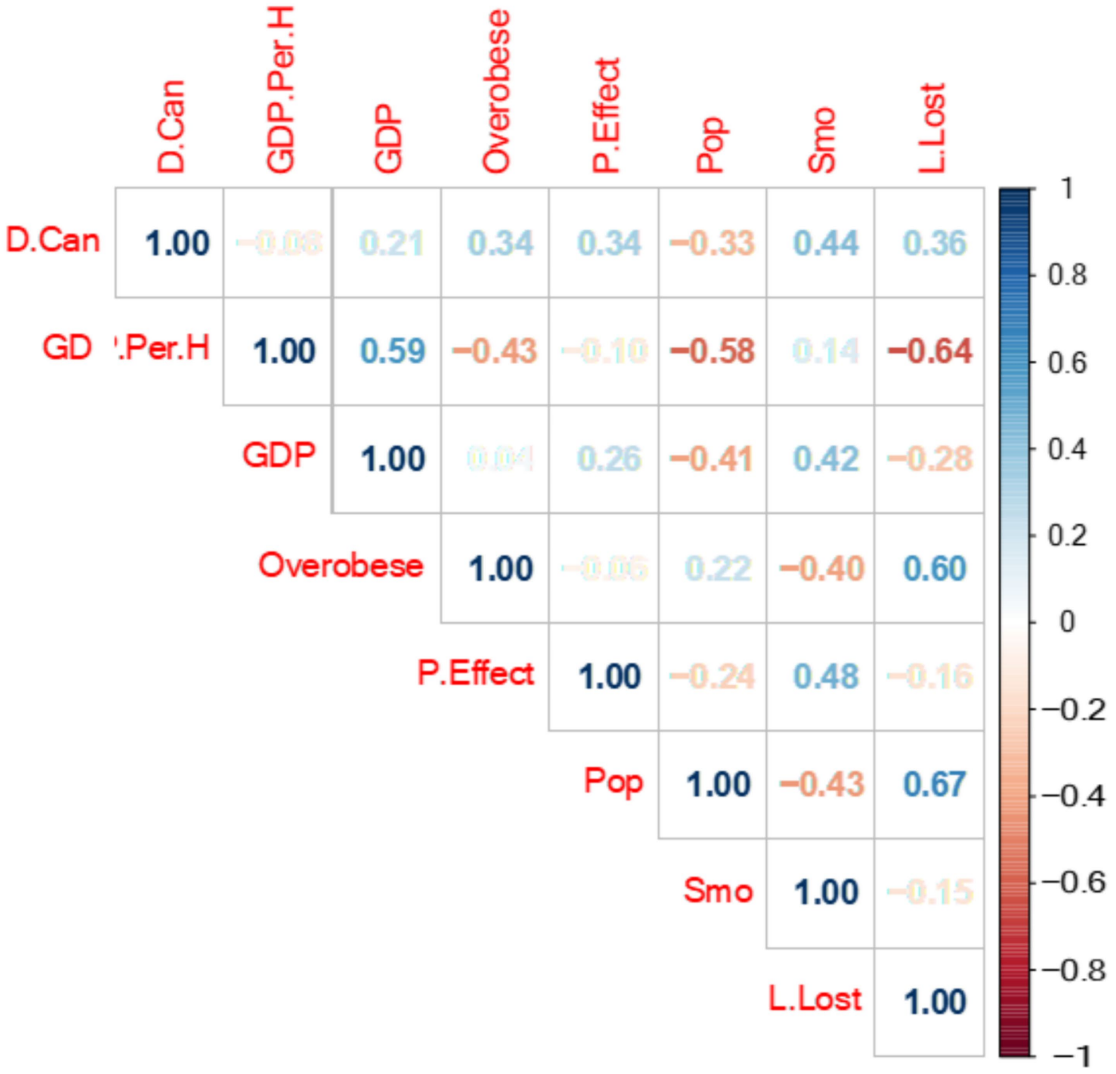
A color palette showing the relationship between dependent and independent variables, namely cancer death rates, pollution effect, smoking, population size, GDP in USD and obesity in G7 countries between the years of 2000 and2020.

Figure 1, a correlation matrix was created showing the relationship between all variables through a color palette. The values in the correlation matrix range from −1 to +1. Values close to −1 indicate a negative correlation, while values close to +1 indicate a positive correlation. Two variables with a positive correlation will increase or decrease together, whereas for two negatively correlated variables, as one increases, the other decreases. A value close to 0 indicates no connection between the two variables.

In consideration of the data given in the table, between 2000 and 2020, there was a positive correlation between cancer deaths and smoking, life loss, pollution effect, obesity, and GDP rates. As these rates increase, the cancer death rate also increases in parallel. The highest positive relationship is with the smoking rate, at 0.44. Therefore, the smoking rate affects the cancer death rate more than the other variables. On the other hand, there is a negative correlation between cancer deaths and population. Thus, as one value increases, the other decreases. For example, as cancer deaths increase, the population decreases, or as the population rises, cancer deaths decrease. The GDP USD per hour worked value is close to 0 (−0.08), indicating no connection between these variables.

The Principal Component Analysis (PCA) reports the factors that are more dominant and illustrative in each country. PCA is a statistical technique universally used in numerous fields, including data visualization, dimensional reduction, data compression, and data analysis. It is specifically convenient for reducing complexity in large and intricate data sets, producing results that make interpretation elementary. This analysis is vital when working with complex and extensive data (16).

**Figure 2:**
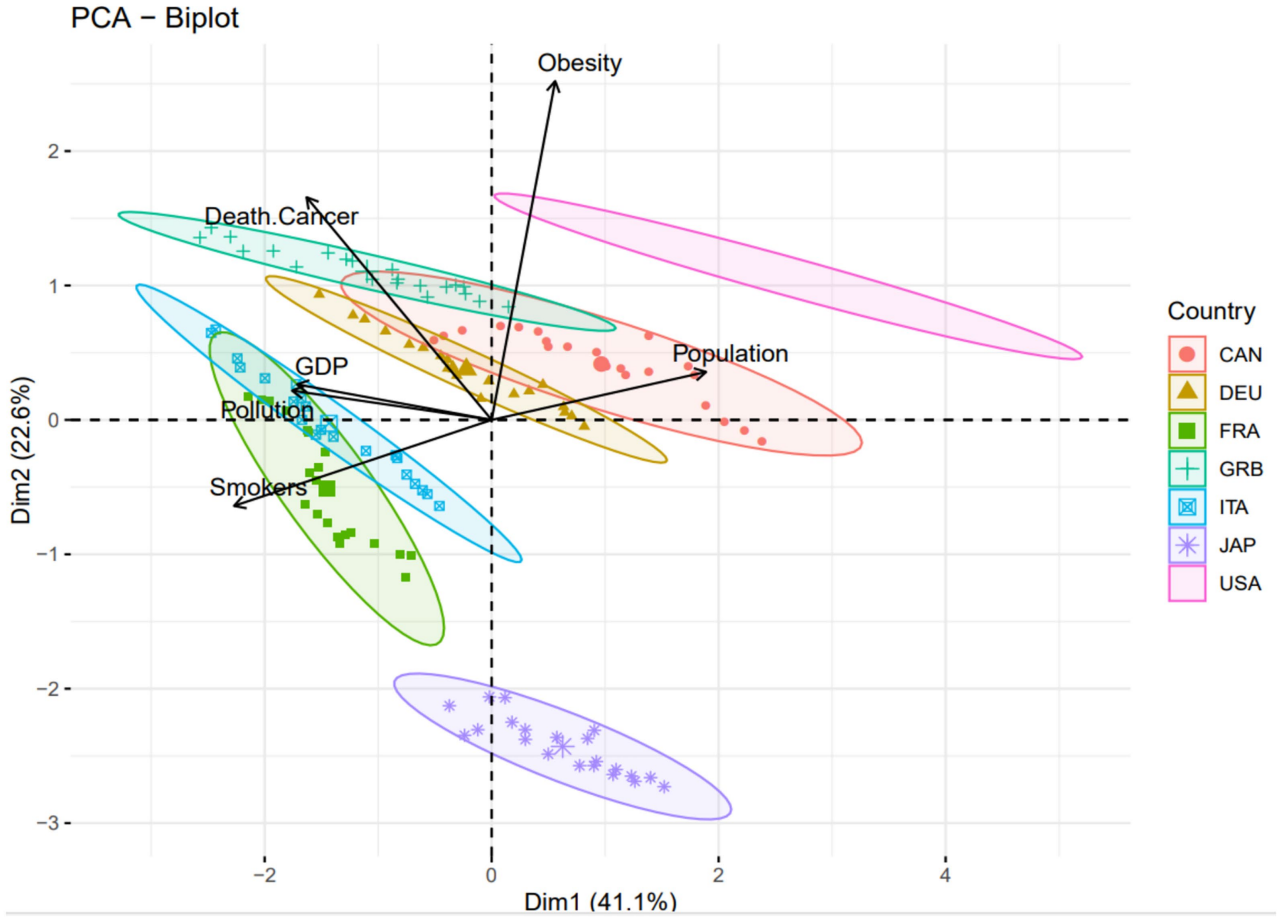
Distribution of dependent and independent variables, namely cancer death rates, pollution effect, smoking, population size, GDP in USD and obesity in G7 countries between the years of 2000 and 2020.

Figure 2 shows the contribution rates of variables to the first (Dim-1) and second (Dim-2) principal components (PCs). About Dim-1 (41.1%), smokers and population variables make the highest contribution, while pollution, GDP, and cancer deaths make moderate contributions. The obesity variable has a relatively lower contribution. This shows that the first component is dominated by smokers and the population. On the other hand, with Dim-2 (22.6%), Obesity and Death Cancer make the highest contribution. Smokers, Population, and GDP have a low effect. Pollution makes the lowest contribution to the second component.

Figure 2 shows that Dim-2 is also closely related to obesity and cancer deaths, but other variables also have a certain effect. In general, the first component (Dim-1) is most associated with smokers and population variables, while the second component is associated with obesity and cancer deaths. This graph shows the directions of the variables and the distribution of the countries. Cancer death and obesity variables appear to be strongly associated with each other (positioned close to the same direction). GDP and Population are close to each other but oriented in a different direction. GDP and pollution appear to be very closely related to each other. Smokers, on the other hand, are looking in a different direction, meaning that they may be more independent of the other variables. Considering the distribution of countries, for instance, the United States (USA) is most likely located in the direction of obesity and population (with the possible high obesity and cancer death rates). Japan (JAP) may be different from the other groups since it probably has low obesity and low cancer death rates. European countries (DEU, FRA, ITA, GRB) may generally be closer to GDP and Population. This biplot analysis is quite significant in terms of visualizing the relationship between countries’ obesity, cancer deaths, smoking rates, and economic indicators. In particular, the strong relationship between obesity and cancer deaths and the fact that the GDP and population are on a different axis are striking.

## Discussion

### Main findings of the study

This study investigated the relationship between various socioeconomic and environmental factors, such as GDP in USD, pollution, population, smoking, years of lives lost (YLL), and obesity and cancer mortality in G7 countries between 2000 and 2020. Among these factors, obesity (*p* < 0.001), GDP (*p* < 0.001), population (*p* < 0.001), environmental pollution levels (*p* < 0.001) and smoking (*p* < 0.001) rates were found to have a significant positive association with cancer mortality.

### Comparison of findings with existing literature and implications

The findings of the current study showed smoking rates are positively correlated with the Cancer deaths that align with existing literature. For instance, a report by the International Agency for Research on Cancer (IARC) published in The Lancet Oncology (2004) confirmed the critical role of smoking as a major risk factor for cancer mortality (17). Wéber et al. (18) similarly emphasized the significant contribution of smoking to cancer deaths. Additionally, Afifi et al. (19) highlighted the relationship between population size and cancer mortality, supporting the current study’s observations.

Evidence has been offered by Bafunno et al.(20) in a review article on the success in Europe of schemes aimed at encouraging the discontinuation of the habit of using tobacco products, which infers that interventions made within the social environment of the public in addition to individual prompting may effectuate a higher level of efficacy. Throughout Europe, yet further influential actions have been taken in the form of educational inducements, the creation of smoke-free environments in public places raised taxes on tobacco products, the placement of health warnings on packaging, and wide-scale media campaigns to encourage behavior change. Since Europe enjoys a thriving economy together with access to large resources, the attainability of reduced cancer deaths through preventative health strategies is realizable. Significantly, it is notable that the public is greatly motivated by incentives and costs to participate in behavior that enhances physical health, incorporating healthier eating models and physical activity into their routines.

Environmental policies targeting pollution also demonstrated significant potential to reduce cancer mortality (21). Between the years 1990 and 2008, pollution was studied by Shapiro and Walker (22) in the USA, concluding that a reduction in pollution in the air was attainable through the execution of enforced environmental regulations. A comparable outcome was also noted in the UK through an analysis carried out by Cole et al. (23). In China studies conducted by Yin et al. (24), Wang and Shen (25), Pei et al. (26), and Song et al. (27) all noted a link between divergent regulations involving the environment and the derived consequences of negative outcomes on levels of CO2 emissions. Similarly, the relationship was comparable in OECD countries according to De Angelis et al. (28), furthermore, substantiation of corresponding outcomes for BRICS countries was reported by Danish et al. (29). Utilizing over 50 years of data from the years 1961 until 2017, Shahzad et al. (30) evaluated the situation in America by using the ecological footprint as an appropriate substitute for existing pollution. The outcome exhibited a conclusive and causal link between the two variables. However, even though it has been noted by Weina et al. (31) that green innovations contribute to environmental improvement productivity, their research in Italy attested that predominantly the CO2 emissions are not greatly reduced.

Furthermore, the findings of this study are parallel to the evidence that shows that socioeconomic disparities increases cancer mortality. Alvarez et al. (32) showed that low-income, low-educated, and minority communities face higher cancer risks due to environmental hazards, emphasizing the need for targeted interventions to reduce these inequalities. Research carried out involving the population of Brazil by Cancela et al. (33) on the consequences of the mortality rate of cancer on individuals of employment age from 2001 to 2030, divulged that economic loss deriving from decreased productivity emerged from atypical types of cancer in young people. Overall, the loss of productivity per death from all types of cancer was ascertained to be very similar in the case of men.

The associations observed in the study emphasize the importance of orchestrated involvement in public health services in the G7 countries. Public Health Programs promoting the reduction in prevalence of tobacco use (34); initiatives and education in methods to decrease obesity prevalence (35), and measures to control causes and effects of harmful consequences of environmental pollution (36) must be prioritized to efficiently prevent risk factors associated with cancer deaths. YLL, an indicator associated with healthcare quality and access to health services, also requires an appreciable consideration. This indicates the mortality rate of cancer patients could be reduced by making improvements in the services towards the prevention of risk factors and promotion of health in populations.

### Limitations of the study

This study investigated the interrelationship between the global increase in cancer deaths and the factors related with environment and economy and lifestyle in seven developed countries (G7 countries) over 20 years. This study is limited to G7 countries, which are developed countries, therefore it would not be possible to generalize the findings to other nations with different socioeconomic, environmental and lifestyle characteristics. The study, furthermore, has limitations sue to using secondary data, which may introduce biases due to the lack of control over the data at the time of the collection. The data used in the study was ecological data that is not based on the characteristics of individual characteristics, but on the characteristics of populations as a whole. The study is, therefore, subject to ecological fallacy. The ecological fallacy occurs when associations recognized at the ecological (population) level data are supposed to be applicable for each individual living in a population group. Ecological fallacy can lead to bias in the findings adopted from regression models; wrong implications to be produced about the interventions by policy makers and may even lead to development of interventions or programs for incorrect group of target populations (37). This study is a retrospective study, limiting causal inferences. Other contextual factors including the impact of healthcare spending (38), genetic predispositions, the exposure of populations to organic pollutants contaminated food products of animal origin, such as hen eggs were not included in the analysis (39). Future studies should explore other contextual factors, including healthcare spending and genetic predispositions to enhance validity.

## Conclusion

This study emphasized the intricate interrelationship between cancer mortality and various environmental, socioeconomic, and lifestyle factors in the group of countries known as the G7 countries. Smoking, obesity, pollution, and the opportunity of healthcare emerged as critical determinants. The outcome highlights the need for comprehensive public health strategies that address these key factors through preventive interventions. By exploiting the available substantial resources in developed nations, policymakers must implement effective smoking cessation programs, promote healthier lifestyles, and adopt stringent environmental regulations. Improving healthcare infrastructure and addressing socioeconomic disparities are also essential to reduce cancer mortality and enhance public health outcomes. Future research should explore the relationships of cancer mortality with various risk factors in populations and focus on developing targeted interventions tailored to these risk factors to achieve sustainable reductions in cancer mortality globally.

## Data Availability

The original contributions presented in the study are included in the article/supplementary material, further inquiries can be directed to the corresponding author.

## References

[1] Dougherty TP Meyer JE. Comparing lifestyle modifications and the magnitude of their associated benefit on Cancer mortality. Nutrients. (2023) 15:2038. doi: 10.3390/nu15092038, PMID: 37432170 PMC10181277

[2] Bravi L Santos G Pagano A Murmura F. Environmental management system according to ISO 14001: 2015 as a driver to sustainable development. Corp Soc Responsib Environ Manag. (2020) 27:2599–614. doi: 10.1002/csr.1985

[3] Ellena M Breil M Soriani S. The heat-health nexus in the urban context: a systematic literature review exploring the socio-economic vulnerabilities and built environment characteristics. Urban Clim. (2020) 34:100676. doi: 10.1016/j.uclim.2020.100676, PMID: 40955376

[4] Lagoa R Marques-da-Silva D Diniz M Daglia M Bishayee A. Molecular mechanisms linking environmental toxicants to cancer development: significance for protective interventions with polyphenols. Semin Cancer Biol. (2022) 80:118–44. doi: 10.1016/j.semcancer.2020.02.002 32044471

[5] Chen SL Nøst TH Botteri E Ferrari P Braaten T Sandanger TM. Overall lifestyle changes in adulthood are associated with cancer incidence in the Norwegian women and cancer study (NOWAC)–a prospective cohort study. BMC Public Health. (2023) 23:633. doi: 10.1186/s12889-023-15476-3, PMID: 37013506 PMC10069035

[6] Miller KD Nogueira L Devasia T Mariotto AB Yabroff KR Jemal A. Cancer treatment and survivorship statistics. CA Cancer J Clin. (2022) 72:409–36. doi: 10.3322/caac.21731, PMID: 35736631

[7] Sanderson SC Waller J Jarvis MJ Humphries SE Wardle J. Awareness of lifestyle risk factors for cancer and heart disease among adults in the UK. Patient Educ Couns. (2009) 74:221–7. doi: 10.1016/j.pec.2008.08.003, PMID: 19059747

[8] Drake I Dias JA Teleka S Stocks T Orho-Melander M. Lifestyle and cancer incidence and mortality risk depending on family history of cancer in two prospective cohorts. Int J Cancer. (2020) 146:1198–207. doi: 10.1002/ijc.32397, PMID: 31077359

[9] Mishra P Pandey CM Singh U Gupta A Sahu C Keshri A. Descriptive statistics and normality tests for statistical data. Ann Card Anaesth. (2019) 22:67–72. doi: 10.4103/aca.ACA_157_18, PMID: 30648682 PMC6350423

[10] Schober P Boer C Schwarte LA. Correlation coefficients: appropriate use and interpretation. Anesth Analg. (2018) 126:1763–8. doi: 10.1213/ANE.0000000000002864, PMID: 29481436

[11] Senaviratna NAMR Cooray TMJA. Diagnosing multicollinearity of logistic regression model. Asian J Probab Stat. (2019) 5:1–9. doi: 10.9734/ajpas/2019/v5i230132

[12] Kumari K Yadav S. Linear regression analysis study. J Pract Cardiovasc Sci. (2018) 4:33–6. doi: 10.4103/jpcs.jpcs_8_18

[13] Elhai JD Calhoun PS Ford JD. Statistical procedures for analyzing mental health services data. Psychiatry Res. (2008) 160:129–36. doi: 10.1016/j.psychres.2007.07.003, PMID: 18585790

[14] Ntumi S. Reporting and interpreting one-way analysis of variance (ANOVA) using a data-driven example: a practical guide for social science researchers. J Res Educ Sci. (2021) 12:38–47. doi: 10.14505/jres.v12.14.04

[15] Balogun AL Tella A. Modelling and investigating the impacts of climatic variables on ozone concentration in Malaysia using correlation analysis with random forest, decision tree regression, linear regression, and support vector regression. Chemosphere. (2022) 299:134250. doi: 10.1016/j.chemosphere.2022.134250, PMID: 35318016

[16] Greenacre M Groenen PJ Hastie T d’Enza AI Markos A Tuzhilina E. Principal component analysis. Nat Rev Methods Primers. (2022) 2:100. doi: 10.1038/s43586-022-00184-w

[17] IARC Working Group on the Evaluation of Carcinogenic Risks to Humans. (2004). Tobacco smoke and involuntary smoking. Lyon: World Health Organization International Agency for Research on Cancer.

[18] Wéber A Morgan E Vignat J Laversanne M Pizzato M Rumgay H. Lung cancer mortality in the wake of the changing smoking epidemic: a descriptive study of the global burden in 2020 and 2040. BMJ Open. (2023) 13:e065303. doi: 10.1136/bmjopen-2022-065303, PMID: 37164477 PMC10174019

[19] Afifi AM Saad AM Al-Husseini MJ Elmehrath AO Northfelt DW Sonbol MB. Causes of death after breast cancer diagnosis: a US population-based analysis. Cancer. (2020) 126:1559–67. doi: 10.1002/cncr.32648, PMID: 31840240

[20] Bafunno D Catino A Lamorgese V Pizzutilo P Di Lauro A Petrillo P. Tobacco control in Europe: a review of campaign strategies for teenagers and adults. Crit Rev Oncol Hematol. (2019) 138:139–47. doi: 10.1016/j.critrevonc.2019.01.022, PMID: 31092369

[21] Kazemzadeh E Fuinhas JA Radulescu M Koengkan M Silva N. The heterogeneous impact of the environmental policy stringency on premature indoor and outdoor deaths from air pollution in the G7 countries: do economic complexity and green innovation matter? Atmos Pollut Res. (2023) 14:101664. doi: 10.1016/j.apr.2023.101664

[22] Shapiro JS Walker R. Why is pollution from US manufacturing declining? The roles of environmental regulation, productivity, and trade. Am Econ Rev. (2018) 108:3814–54. doi: 10.1257/aer.20151272

[23] Cole MA Elliott RJ Shimamoto K. Industrial characteristics, environmental regulations and air pollution: an analysis of the UK manufacturing sector. J Environ Econ Manag. (2005) 50:121–43. doi: 10.1016/j.jeem.2004.08.001

[24] Yin J Zheng M Chen J. The effects of environmental regulation and technical progress on CO2 Kuznets curve: an evidence from China. Energy Policy. (2015) 77:97–108. doi: 10.1016/j.enpol.2014.11.008

[25] Wang Y Shen N. Environmental regulation and environmental productivity: the case of China. Renew Sust Energ Rev. (2016) 62:758–66. doi: 10.1016/j.rser.2016.05.048

[26] Pei Y Zhu Y Liu S Wang X Cao J. Environmental regulation and carbon emission: the mediation effect of technical efficiency. J Clean Prod. (2019) 236:117599. doi: 10.1016/j.jclepro.2019.07.074

[27] Song M Wang S Zhang H. Could environmental regulation and R&D tax incentives affect green product innovation? J Clean Prod. (2020) 258:120849. doi: 10.1016/j.jclepro.2020.120849

[28] De Angelis EM Di Giacomo M Vannoni D. Climate change and economic growth: the role of environmental policy stringency. Sustainability. (2019) 11:2273. doi: 10.3390/su11082273

[29] Danish Ulucak R Khan SUD Baloch MA Li N. Mitigation pathways toward sustainable development: Is there any trade-off between environmental regulation and carbon emissions reduction? Sustain Dev. (2020) 28:813–22. doi: 10.1002/sd.2032

[30] Shahzad U Fareed Z Shahzad F Shahzad K. Investigating the nexus between economic complexity, energy consumption and ecological footprint for the United States: new insights from quantile methods. J Clean Prod. (2021) 279:123806. doi: 10.1016/j.jclepro.2020.123806

[31] Weina D Gilli M Mazzanti M Nicolli F. Green inventions and greenhouse gas emission dynamics: a close examination of provincial Italian data. Environ Econ Policy Stud. (2016) 18:247–63. doi: 10.1007/s10018-015-0126-1

[32] Alvarez CH Evans CR. Intersectional environmental justice and population health inequalities: a novel approach. Soc Sci Med. (2021) 269:113559. doi: 10.1016/j.socscimed.2020.113559, PMID: 33309156

[33] Cancela MDC Dos Santos JEM de Souza LBL Martins LFL de Souza DLB Barchuk A. The economic impact of cancer mortality among working-age individuals in Brazil from 2001 to 2030. Cancer Epidemiol. (2023) 86:102438. doi: 10.1016/j.canep.2023.102438 37579673 PMC10577440

[34] Shiels MS Graubard BI McNeel TS Kahle L Freedman ND. Trends in smoking-attributable and smoking-unrelated lung cancer death rates in the United States, 1991-2018. JNCI J Natl Cancer Inst. (2024) 116:711–6. doi: 10.1093/jnci/djad256, PMID: 38070489 PMC11077306

[35] Pati S Irfan W Jameel A Ahmed S Shahid RK. Obesity and cancer: a current overview of epidemiology, pathogenesis, outcomes, and management. Cancer. (2023) 15:485. doi: 10.3390/cancers15020485, PMID: 36672434 PMC9857053

[36] Gatti RC Di Paola A Monaco A Velichevskaya A Amoroso N Bellotti R. The spatial association between environmental pollution and long-term cancer mortality in Italy. Sci Total Environ. (2023) 855:158439. doi: 10.1016/j.scitotenv.2022.158439 36113788

[37] Li Q. Assessing and adjusting for bias in ecological analysis using multiple sample datasets. BMC Med Res Methodol. (2025) 25:112. doi: 10.1186/s12874-025-02552-y, PMID: 40275196 PMC12023363

[38] Coles E Anderson J Maxwell M Harris FM Gray NM Milner G. The influence of contextual factors on healthcare quality improvement initiatives: a realist review. Syst Rev. (2020) 9:1–22. doi: 10.1186/s13643-020-01344-3, PMID: 32336290 PMC7184709

[39] Giannico OV Baldacci S Basile FC Pellegrino A Desiante F Franco E. PCDD/fs and PCBs in hen eggs from a contaminated area in Italy: a 9 years spatio-temporal monitoring study. Food Addit Contam Part A Chem Anal Control Expo Risk Assess. (2023) 40:294–304. doi: 10.1080/19440049.2022.2157051, PMID: 36602427

